# Cardiotoxicity of Epidermal Growth Factor Receptor 2-Targeted Drugs for Breast Cancer

**DOI:** 10.3389/fphar.2021.741451

**Published:** 2021-11-01

**Authors:** ZiYan Yang, Wei Wang, Xiaojia Wang, ZhiQuan Qin

**Affiliations:** ^1^ Department of Oncology Center, Oncology, Zhejiang Provincial People’s Hospital, People’s Hospital of Hangzhou Medical College, Hangzhou, China; ^2^ Graduate School of Bengbu Medical College, Bengbu, China; ^3^ Department of Breast Medical Oncology, Cancer Hospital of the University of Chinese Academy of Sciences (Zhejiang Cancer Hospital), Hangzhou, China

**Keywords:** cardiotoxicity, ErbB2, targeting drugs, breast cancer, therapy

## Abstract

Breast cancer is the most common form of cancer in women and its incidence has been increasing over the years. Human epidermal growth factor receptor 2 (HER2 or ErbB2) overexpression is responsible for 20 to 25% of invasive breast cancers, and is associated with poor prognosis. HER2-targeted therapy has significantly improved overall survival rates in patients with HER2-positive breast cancer. However, despite the benefits of this therapy, its cardiotoxicity is a major concern, especially when HER2-targeted therapy is used in conjunction with anthracyclines. At present, the mechanism of this cardiotoxicity is not fully understood. It is thought that HER2-targeting drugs inhibit HER2/NRG 1 dimer formation, causing an increase in ROS in the mitochondria of cardiomyocytes and inhibiting the PI3K/Akt and Ras/MAPK pathways, resulting in cell apoptosis. Antioxidants, ACE inhibitors, angiotensin II receptor blockers, β-blockers, statins and other drugs may have a cardioprotective effect when used with ErbB2-targeting drugs. NT-proBNP can be used to monitor trastuzumab-induced cardiotoxicity during HER2-targeted treatment and may serve as a biological marker for clinical prediction of cardiotoxicity. Measuring NT-proBNP is non-invasive, inexpensive and reproducible, therefore is worthy of the attention of clinicians. The aim of this review is to discuss the potential mechanisms, clinical features, diagnostic strategies, and intervention strategies related to cardiotoxicity of ErbB2-targeting drugs.

## 1 Introduction

Breast cancer is the most common cancer among women worldwide, and its incidence has been increasing yearly (Bray et al., 2018). Chemotherapy is one of the main treatments for breast cancer (Piccart-Gebhart and Sotiriou, 2007). Human epidermal growth factor receptor 2 (HER2), also known as erythroblast leukemia virus oncogene homolog 2 (ErbB2), is overexpressed in 20–25% of breast cancers. This transmembrane receptor promotes abnormal cell growth and proliferation in human breast cancer, leading to tumor cell invasion and poor prognosis (Slamon et al., 1987). HER2/ErbB2 are potential targets in chemotherapy of HER2-positive (HER2+) breast cancer. The 2021 ASCO Guidelines indicated that ErbB2-targeting drugs significantly improved survival rates and more patients were included in the range of drug (Korde et al., 2021). Unfortunately, target drugs is often discontinued once cardiotoxicity occurs during clinical (Perez and Rodeheffer, 2004). Cardiotoxicity is mainly caused by the reversible decrease of ejection fraction, but also severe heart failure and even fatal (Jerusalem et al., 2019).

**FIGURE 1:**
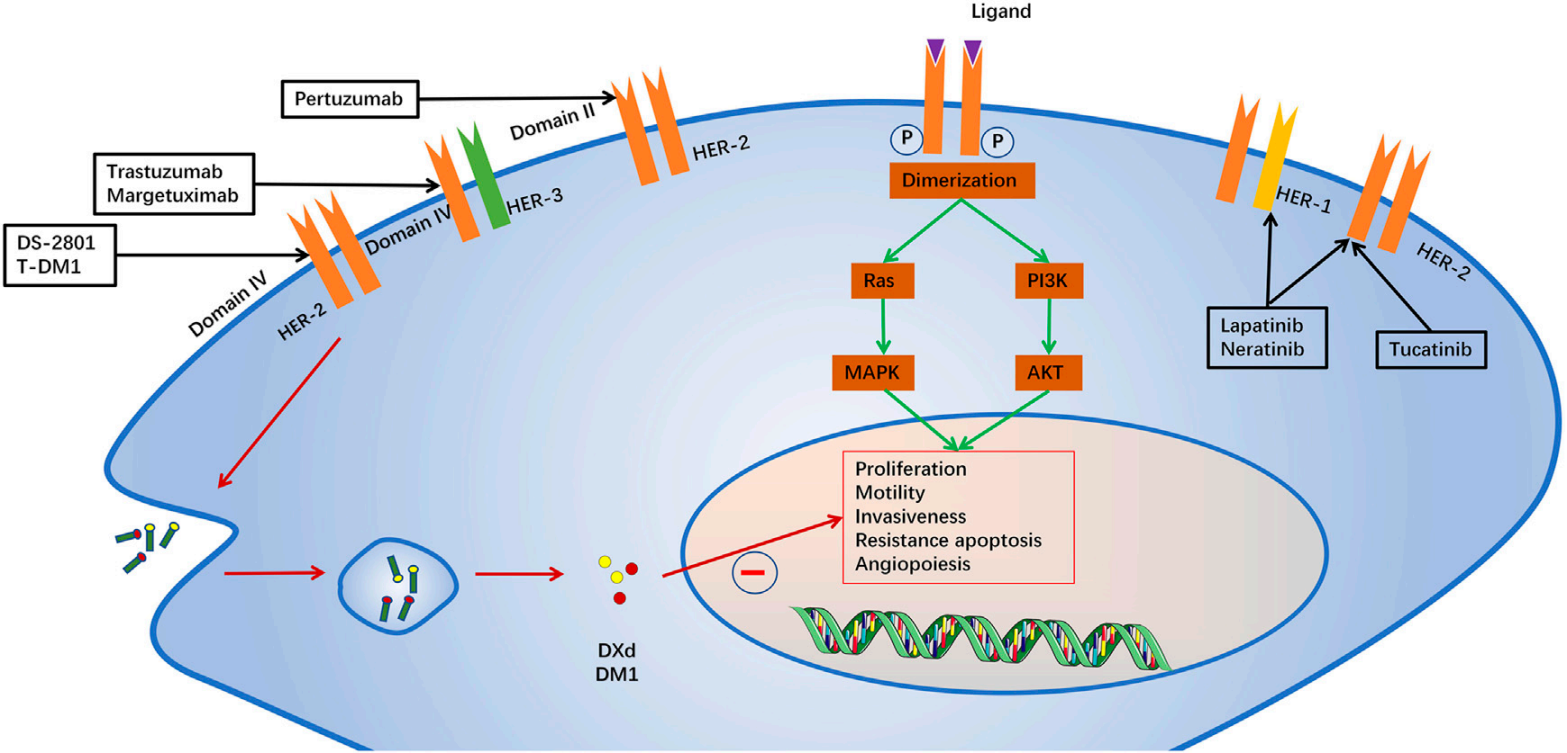
Mechanism of action of ErbB2-targeted drugs.

HER2 belongs to a family of receptor tyrosine kinases with four members: HER1 (EGFR), HER2, HER3 and HER4. When activated, the HER proteins homodimerize or heterodimerize and subsequently activate intricate cellular signalling cascades, including the PI3K/AKT and RAS/MAPK (ERK) pathways, which regulate cell proliferation and survival, as well as the metastasis of tumour cells (Slamon et al., 1987). ErbB2-targeted drugs include monoclonal antibodies, antibody drug conjugates and tyrosine kinase inhibitors (Godeau et al., 2021). Monoclonal antibodies mainly include trastuzumab, pertuzumab and margetuximab. Trastuzumab, a humanized monoclonal antibody against ErbB2 domain IV was the first immunotherapeutic agent for HER2(+) breast cancer (Jerian and Keegan, 1999). Margetuximab is a novel anti-HER2 antibody that has a higher affinity with the Fc receptor and stronger antibody-dependent cell-mediated antitumor cytotoxicity (ADCC) (Kaplon and Reichert, 2021). Pertuzumab is another humanized monoclonal antibody that binds to ErbB2 domain II and inhibits its dimerization (Capelan et al., 2013). Trastuzumab emtansine(T-DM1) and Trastuzumab deruxtecan (DS-2801,Enhertu) are antibo-cytotoxic drug conjugates composed of trastuzumab with the microtubule toxin DM1 and topoisomerase I inhibitor, a potent mitotic inhibitor (Zhao et al., 2020; Andrikopoulou et al., 2021). T-DM1 is currently a second-line treatment for patients with metastatic HER2(+) breast cancer (Verma et al., 2012). The Tyrosine kinase inhibitors include lapatinib, Neratinib and Tucatinib (Chaar et al., 2018). Lapatinib is an oral tyrosine kinase inhibitor that reverses ErbB2 and endothelium growth factor receptor (EGFR orErbB1) signaling (Moy et al., 2007). Neratinib is an irreversible small molecule inhibitor of HER1, HER2 and HER4 tyrosine kinases, approved for the extended adjuvant treatment of women with early-stage and metastatic HER2 + breast cancer (Oh and Bang, 2020). Tucatinib, a newly approved tyrosine kinase inhibitor, is characterized by its high selectivity for HER2/ErbB2 (Corti and Criscitiello, 2021). In order to better understand the cardiotoxicity of ErbB2-targeted drugs, we have systematically reviewed recently published papers on the potential mechanisms, clinical manifestations, diagnostic strategies, intervention strategies, and the latest progress in ErbB2-targeted drug cardiotoxicity. We summarized the potential mechanism and intervention strategies with ErbB2/nauregulin 1 (NRG1) pathway causing cardiac dysfunction reported to date, to provide more evidence for clinical practice (Figure 1).

## 2 Mechanism of Cardiotoxicity

The ErbB receptor is a transmembrane receptor tyrosine kinase that regulates cell physiological responses including cell growth, division, differentiation, adhesion, function, and apoptosis (Linggi and Carpenter., 2006). ErbB signaling in the heart is critical for the normal development of the fetal heart (Gassmann et al., 1995). In mutant mice with a deletion of the ErbB2 gene, abnormal ventricular trabeculae resulted in fetal death (Lee et al., 1995; Meyer and Birchmeier, 1995). In addition, ErbB2 plays an important role in adult cardiomyocytes growth (Zhao et al., 1998). ErbB2 mutant mice showed decreased ErbB2 expression and impaired ventricular dilation and contraction, and histology of the myocardium revealed ultrastructural changes (Ozcelik et al., 2002). Trastuzumab and pertuzumab reduced the dimerization of ErbB2/4 in rat and human cardiomyocytes (Fedele et al., 2012a). The NRG-1/ErbB2/ErbB4 complex controls cardiomyocyte survival and myofibrillary disorders in cardiomyocytes (Kuramochi et al., 2006). NRG-1 activation directly promotes cardiomyocyte survival through the ErbB2/ERBB4 heterodimer (De Keulenaer et al., 2010). NRG1 activates PI3-kinase/Akt and MAPK/Erk1/2 pathways through ErbB2 phosphorylation (Lemmens et al., 2004). Silencing or down-regulation of ErbB2 expression attenuated NRG-1-induced intracellular Akt and ERK1/2 phosphorylation (Hsu et al., 2018).

**FIGURE 2:**
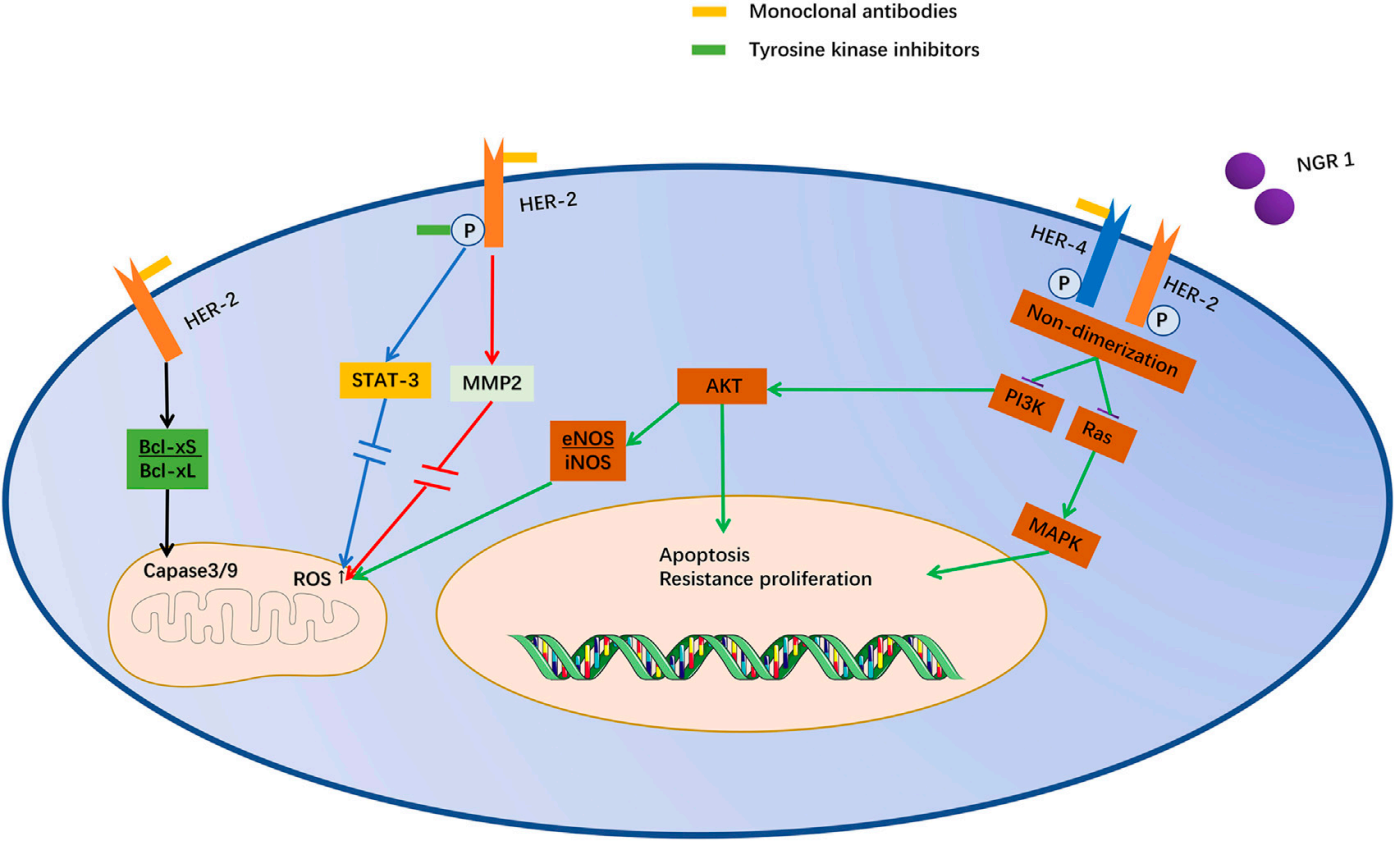
Potential mechanism of cardiotoxicity induced by anti-ErbB-targeted drugs. Trastuzumab and T-DM1 act on the same epitope of ErbB2, and pertuzumab also acts on similar epitopes. They inhibit ErbB2/4 dimerization induced by NRG1. Lapatinib acts directly on the phosphorylation site of ErbB2. Inhibition of ErbB2 can inhibit STAT3 through MAPK/ERK1/2, leading to mitochondrial dysfunction and promoting cell death. Moreover, Akt expression can decrease iNOS and increase eNOS, which leads to the accumulation of ROS in mitochondria. Moreover, the increase in the Bcl-XS/Bcl-XL ratio directly stimulates caspase 3/9 and promotes cell apoptosis.

NRG1 is a ligand of the epidermal growth factor family, which can bind to ErbB3 or ErbB4 monomers and induce the formation of homodimers (ErbB4/4) and heterodimers (ErbB2/3 or ErbB2/4) (Lemmens et al., 2007). The NRG1 stimulates glucose uptake and protein synthesis in cardiomyocytes. (Cote et al., 2005). ErbB2 inhibition decreased expression of endothelial nitric oxide synthase (eNOS) and increased inducible nitric oxide synthase(iNOS), leading to produce more reactive oxygen species (ROS) (Timolati et al., 2006). NRG1 reduces contraction without impairing diastole by upregulating NOS and reducing the effect of β-adrenergic stimulation (Lemmens et al., 2004). STAT-3 is a transcription factor that is activated by tyrosine phosphorylation in response to certain ligand, such as interferon and epidermal growth factor (Tkach et al., 2013). It plays a key role in cell growth and differentiation, leading to ultrastructural changes in cardiomyocytes (Kabel and Elkhoely, 2017). ErbB2 inhibition can lead to increased Bcl-xS/Bcl-xL ratio, activation of mitochondrial caspase-9 and caspase-3, and causing apoptosis (Rohrbach et al., 2005). ErbB2 inhibition has also been reported to alter Bcl-x splicing, induce endogenous apoptotic signaling (Grazette et al., 2004; De Lorenzo et al., 2018). Besides, Matrix metalloproteinase-2 (MMP2) mRNA is elevated in trastuzumab cardiotoxicity, accompanied by an increase in ROS (Riccio et al., 2018). Lapatinib can affect cardiac function and fibrosis in mice (Fedele et al., 2012b). Lapatinib can directly inhibit ErbB2 phosphorylation (Sawyer et al., 2002). Although lapatinib did not affect NOS expression and basal mitochondrial respiration, it impaired the standby oxygen consumption rate (Hsu et al., 2018). Cardiotoxicity caused by the inhibition of ErbB2 may be due to the adaptation of the heart to stress reactions. There is evidence that trastuzumab, pertuzumab, and lapatinib reduce cell viability in a concentration-dependent manner (Fedele et al., 2012a). Furthermore, ErbB2 inhibition increased ROS production and impaired mitochondrial function in a concentration-dependent manner (Pentassuglia et al., 2007) (Figure 2).

## 3 Clinical Features of Cardiotoxicity

### 3.1 Monoclonal Antibody

The main reason for discontinuation of ErbB2-targeted therapy is cardiotoxicity (Martín et al., 2009). Trastuzumab was the first ErbB2-targeting drug to be used in HER + breast cancer, therefore, trastuzumab-associated cardiotoxicity is the most well-studied cardiotoxicity of the cardiotoxicities associated with Erb2-targeting drugs (Herrmann, 2020). Trastuzumab-associated cardiotoxicity is usually characterized by an asymptomatic decrease in left ventricular ejection fraction (LVEF), which can be reversed after drug discontinuation (Perez and Rodeheffer, 2004). However, a prospective study showed that 48.53% of patients with available cardiac ultrasound measurements (379 out of 781 patients) did not fully recover baseline LVEF (Jacquinot et al., 2018). Nonetheless, more than 30% of the people in the study were 60 years old. Therefore, the failure to exclude elderly patients with heart diseases from the study may have caused bias. Unfortunately, the study did not conduct a follow up, so long-term LVEF recovery results were not available. However, Yoon et al. found that non-recovery of trastuzumab-induced left ventricular dysfunction (LVD) had an impact on the clinical outcome of breast cancer. The survival rates of the group without left ventricular hypertrophy were significantly lower than those of the group with left ventricular hypertrophy. Increased left ventricular volume, pulmonary hypertension, and anemia were found to be contributing factors (Yoon et al., 2019). A recent study indicated that patients with reduced baseline cardiac function undergoing trastuzumab therapy for breast cancer developed symptomatic heart failure more frequently than patients with normal cardiac function, but did not experience a higher risk of LVEF decline (Nowsheen et al., 2018). These results contradict a previous study by Romond et al. (2012), that detected LVEF decline during trastuzumab treatment. However, the latter study tracked patients for up to 5 years and this may be the main reason for the opposing results. The development of diastolic dysfunction after treatment with anthracyclines alone, or anthracycline plus trastuzumab, is common (Serrano et al., 2015). However, the development of diastolic dysfunction was not observed with trastuzumab alone (Upshaw et al., 2020). Trastuzumab may cause right heart failure and right ventricular dysfunction and its effect on myocardial function was global and uniform (Bayar et al., 2015; Keramida et al., 2019). Hussain et al. found that patients with asymptomatic LVEF decline to <50% continued to use trastuzumab, who are expected to benefit from additional anti-HER2 therapy (Hussain et al., 2019).

Pertuzumab, in combination with other drugs, mostly causes neutropenia and diarrhoea. Significant cardiac toxicity is rare with both regimens, and overall toxicity is manageable (van Ramshorst et al., 2016). Tan et al. (2021) obtained the same result and the occurrence of cardiotoxic events was less than 1%. Lynce et al. (2019) observed no statistical difference in the incidence of adverse cardiac events between pertuzumab combined with trastuzumab and trastuzumab alone. This was consistent with the results of two previous prospective studies (von Minckwitz et al., 2017). Pertuzumab may only inhibit ErbB2/3 dimerization but does not block the ErbB2/4 signalling pathway in cardiomyocytes (Franklin et al., 2004).

The safety of margetuximab combined with chemotherapy was considered to be acceptable, and margetuximab improved primary progression-free survival (PFS) compared with trastuzumab, with a 24% relative risk reduction (Rugo et al., 2021). In December 2020, margetuximab was approved by the United States Food and Drug Administration (FDA) for use in combination with chemotherapy for metastatic HER2+ breast cancer (Markham, 2021). Treatment was well-tolerated, with toxicities mostly consisting of constitutional symptoms such as diarrhea, nausea, anemia, and pyrexia. A phase I study found no LVEF reduction to <50% or symptomatic heart failure with the use of margetuximab (Bang et al., 2017). Primary analysis of results of the phase III Sophia trial, reported that the incidence of LVEF of any grade was lower in the margetuximab group, than in the trastuzumab group (Rugo et al., 2019).

### 3.2 Antibody–Drug Conjugates

The incidence of cardiac events (CEs) was low in patients treated with trastuzumab emtansine (T-DM1) (Krop et al., 2014). The latest meta-analysis included individual patient-level data of 1,961 patients exposed to T-DM1 from seven trials. Multivariate analysis showed age ≥65 years (OR 3.0; 95% CI, 1.77–5.14; *p* < 0.001) and baseline LVEF <55% (OR 2.62; 95% CI, 1.29–5.32; *p* = 0.008) as risk factors. The majority (79%) of patients had CE resolution after discontinuation of treatment (Pondé et al., 1990). The Phase III Marianne trial compared T-DM1 to T-DM1 + pertuzumab and trastuzumab + taxane, and both T-DM1-containing regimens (0.8 and 2.5%, respectively) had a lower incidence of LVEF reduction than the trastuzumab regimen (4.5%) (Perez et al., 2017). In the Katherine trial, cardiac adverse events were very rare overall (0.3%), but the incidence of T-DM1 (1 in 740) was still lower than that of trastuzumab (4 per 720) (von Minckwitz et al., 2019).

Trastuzumab deruxtecan (DS-8201) was approved by the FDA for the treatment of unresectable or metastatic HER2-positive breast cancer in December 2019 (Narayan et al., 2021). Trastuzumab deruxtecan rarely causes cardiotoxic events, and the most common adverse effects are hematological, including anemia, neutropenia, thrombocytopenia, and leukopenia. Other adverse effects include interstitial lung disease and pneumonia (Modi et al., 2020).

### 3.3 Tyrosine Kinase Inhibitors

Lapatinib is well tolerated and has a low incidence of cardiotoxicity, with mild diarrhea and rash being the most common toxic effects (Bilancia et al., 2007; de Azambuja et al., 2014). Eiger et al. (2020) discovered that compare trastuzumab (T) with galapatinib (L) dual HER2-blocking treatment to trastuzumab, CE was observed in 363 (8.6%) and 166 (7.9%) patients in the T + L arm versus 197 (9.3%) in the T arm (OR = 0.85; [95% CI, 0.68–1.05]).

Neratinib had a bigger problem—diarrhoea in clinical (Chan et al., 2016). In the Nefert-T study, the incidence of grade 3 or higher cardiotoxicity was 1.3% in the neratinib/paclitaxel group and 3.0% in the trastuzumab/paclitaxel group (Awada et al., 2016). In the Extenet trial, and no long-term cardiovascular toxicity was observed. Although cardiotoxicity is negligible, other obvious adverse events, such as diarrhea, require clinician attention (Martin et al., 2017).

Diarrhea and hepatotoxicity were reported as the major adverse events of tucatinib (Lee, 2020). On April 2020, the FDA approved tucatinib in combination with trastuzumab and capecitabine for the treatment of patients with advanced unresectable or metastatic HER2-positive breast cancer, including patients with brain metastases (Shah et al., 2021). In the HER2CLIMB phase III trial, cardiotoxicity was less than 1% in both groups of participants (Murthy et al., 2020).

The latest individual patient data level pooled analysis of HERA, NSBAP B-31, and NCCTG 9831 (Alliance Trials) revealed baseline risk factors that were significantly associated with the development of CE. These factors were baseline LVEF <60%, hypertension, body mass index > 25, age ≥ 60 years, and non-Caucasian ethnicity (de Azambuja et al., 2020). In addition, Jones et al. (2018) found that cardiac function in the first 3 months after trastuzumab treatment had an impact on the long-term assessment of heart failure (6–24 months after treatment), and patients with no significant decrease in EF at 3 months tended to have better long-term assessment of heart failure. The French national multicentre prospective CANTO (CANcer TOxicities) study showed that obesity appears to be associated with an important increase in risk-related cardiotoxicity, which is consistent with the results of meta-analysis (Kaboré et al., 2019) (Tables 1,2).

**TABLE 1:** Summary of studies for development of clinical HER-2 target drugs.

Author/Date	Trial type	Population studies	Number	Methods	Significant	References
Jacquinot et al. (2018)	Prospective study	Patients who received 12 months of trastuzumab	1631	LVEF performed every 3 months; every 6 months (patients received trastuzumab and after completion of treatment over the first 2 years)	48.53% of patients with available measures did not fully recover their baseline LVEF value	Jacquinot et al. (2018)
Yoon et al. (2019)	Retrospective study	Patients with trastuzumab-induced left ventricular dysfunction (LVD)	243	Major adverse clinical events (MACEs) were compared in non-recovered LVD and recovered LVD.	Non-recovered LVD was associated with MACEs. Decreased LVEF, enlarged LV size, pulmonary hypertension, and anaemia were independent predictors of LV-functional non-recovery	Yoon et al. (2019)
Nowsheen et al. (2018)	Retrospective study	Patients with reduced left ventricular ejection fraction during using trastuzumab	428	A retrospective study of women treated with trastuzumab for human epidermal growth factor receptor 2 breast cancer at Mayo Clinic Rochester between January 1, 2000 and August 31, 2015 with pre- and on-therapy echocardiograms available for review	Impaired baseline cardiac function experience no higher risk of LVEF decline, but more frequently develop symptomatic heart failure	Nowsheen et al. (2018)
Hussain et al. (2019)	Retrospective study	Patients with reduced left ventricular ejection fraction during using trastuzumab	160	Retrospectively studied 160 patients with breast cancer receiving trastuzumab in the adjuvant (*n* = 129) as well as metastatic (*n* = 31) settings in our institution from 2006 to 2015. During the median follow-up of 3.5 years	Lower LVEF before trastuzumab independently predicted subsequent development of TRC	Hussain et al. (2019)
Kaboré et al. (2019)	Prospective study	Patients with stage I–III BC treated with anthracycline and/or trastuzumab	929	Analyzed associations between BMI and cardiotoxicity using multivariate logistic regression	The obese group was more prone to cardiotoxicity than the normal-weight group. Obesity and administration of trastuzumab were independently associated with c ardiotoxicity	Kaboré et al. (2019)
Keramida et al. (2019)	Prospective study	Patients with consecutive receiving trastuzumab for 12 months	101	Comprehensive two-dimensional echocardiography with speckle tracking imaging of LV and RV global longitudinal strain (GLS) and RV free wall longitudinal strain (FWLS) analyses were performed at baseline and every 3 months up to treatment completion	Deformation mechanics of both the left and right ventricle follow similar temporal pattern and degree of impairment, confirming the global and uniform effect of trastuzumab on myocardial function	Keramida et al. (2019)

**TABLE 2:** Summary of studies for toxicities of HER-2 target drugs.

ERBb2-Targeted drugs		Toxicities	References
Monoclonal antibody	Trastuzumab	LVEF decreased, HF happened, Arrhythmia	Romond et al. (2012), Bayar et al. (2015), Serrano et al. (2015), Jacquinot et al. (2018), Nowsheen et al. (2018), Hussain et al. (2019), Keramida et al. (2019), Yoon et al. (2019), Upshaw et al. (2020)
	Pertuzumab	Neutropenia and diarrhoea	van Ramshorst et al. (2016), Tan et al. (2021)
	Margetuximab	Diarrhoea, nausea, anaemia and pyrexia	Markham (2021)
Antibody–drug conjugates	T-DM1	Transient LVEF decreased	(Krop et al. (2014), Pondé et al. (1990), Perez et al. (2017), von Minckwitz et al. (2019)
	DS-8201	Anaemia, neutropenia, thrombocytopenia, leukopenia, and interstitial lung disease or pneumonia	Modi et al. (2020)
Tyrosine kinase inhibitors	Lapatinib	Mild diarrhoea and rash	Bilancia et al. (2007), de Azambuja et al. (2014), Eiger et al. (2020)
	Neratinib	Diarrhoea	Awada et al. (2016), Chan et al. (2016), Martin et al. (2017)
	Tucatinib	Diarrhoea and hepatotoxicity	Lee (2020), Shah et al. (2021)

LVEF, left ventricular ejection fraction; HF, heart failure; T-DM1, Trastuzumab emtansine; DS-8201, Trastuzumab deruxtecan.

## 4 Diagnostic Strategies

### 4.1 Imaging

Cardiac ultrasonography is the main method to detect heart failure caused by cardiotoxicity (Fallah-Rad et al., 2011). Impairment of the left ventricular diastolic function before treatment is an independent predictor of trastuzumab cardiotoxicity, and assessment of diastolic function before administration predicts cardiotoxicity risk (Cochet et al., 2011). Moreover, diastolic dysfunction was more sensitive than left ventricular ejection fraction on radiographic examination (Cao et al., 2015). Global longitudinal strain (GLS) analysis can detect cardiac changes earlier and more comprehensively (Lorenzini et al., 2017). A retrospective study showed that anthracycline trastuzumab treatment resulted in early worsening of left ventricle GLS, peripheral strain, and systolic strain rate and the right ventricle GLS and strain rate are also affected. However, early changes in GLS are a good predictor of cardiotoxicity (Arciniegas Calle et al., 2018). GLS based on the 3-apex viewpoint is the preferred technique for detecting cardiac toxicity (Ben Kridis et al., 2020). The latest meta-analysis, including 21 studies comprising of 1782 patients treated with anthracyclines with or without trastuzumab, found the high-risk cut-off values ranged from −21.0 to −13.8%, with worse GLS associated with a higher cancer therapy-related cardiac dysfunction (CTRCD) risk (odds ratio, 12.27; 95% CI, 7.73–19.47; area under the HSROC, 0.86; 95% CI, 0.83–0.89) (Oikonomou et al., 2019). Patients with persistent worsening in diastolic function while taking breast cancer chemotherapeutic agents have a small risk of subsequent systolic dysfunction (Upshaw et al., 2020). There are two new prospective studies comparing variability of echocardiography and cardiovascular magnetic resonance (CMR) in detecting cardiac dysfunction associated with cancer chemotherapy, but the results of these studies are inconclusive (Lambert et al., 2020; Houbois et al., 2021). Therefore, 2D-GLS appears to be the most suitable for clinical applications in individual patients.

### 4.2 Biological Markers

With increasing research, the detection of cardiotoxicity is not limited to imaging, and the use of biological markers is becoming more common in clinical practice (Upshaw, 2020). Placental growth factor (PLGF), growth differentiation factor 15 (GDF-15), high-sensitivity C-reactive protein (hs-CRP), myeloperoxidase (MPO), and troponin I (TnI) can predict decreased LVEF and are promising biomarkers for detecting cardiac function (Onitilo et al., 2012; Bonnie et al., 2014; Putt et al., 2015). A sub-study of the NEOALTTO trial suggested troponin T (TnT) and the amino-terminal fragment of brain natriuretic peptide (NT-proBNP) do not provide an early predictor of cardiac toxicity (Ponde et al., 2018). Besides, a meta-analysis found that an increase in the average BNP/NT-proBNP level of patients after treatment cannot predict left ventricle dysfunction (Michel et al., 2020). But, the latest prospective study, the NEOALTTO trial, fifty newly diagnosed human epidermal growth factor receptor 2-positive BC women received or did not receive anthracycline followed by taxus and trastuzumab for 15 months of follow-up, found NT-pro-BNP measured at the completion of anthracyclines are useful in the prediction of subsequent TIC (Ben Kridis et al., 2020). The NEOALTTO trial had only 11 study patients and receiving trastuzumab and lapatinib two targeted therapies may be the factors. Moreover, the meta-analysis research object is all tumor patients, not just breast cancer and BNP is more susceptible compared to NT-proBNP. The circulating level of NT-proBNP is increased in the unselected cancer patient population, which is related to the increase of myocardial performance index (MPI) value, and is closely related to all-cause mortality (Yildirim et al., 2013; Pavo et al., 2015). GeparOcto-GBG 84 Trial also found a small but significant increase in early NT-proBNP levels in patients with cardiotoxic reactions. NT-proBNP and haemoglobin were significantly associated with cardiotoxicity in patients receiving dose-intensive chemotherapy for early-stage breast cancer, whereas hypersensitive cardiac troponin T was not (Rüger et al., 2020). This may be because direct necrosis of the heart tissue results in more cardiomyocyte dysfunction, as well as the short half-life of TnI and systematic errors caused by experimental design and detection technology. Andersson et al. (2021) found the sensitivity and specificity of NT-proBNP in the detection of trastuzumab induced cardiotoxicity (TIC) were 100 and 95% and changes in NT-proBNP may be used to monitor TIC in patients receiving trastuzumab treatment. They also provide a prognostic value (Pudil et al., 2020). Therefore, NT-proBNP level is an indicator worthy of clinical attention.

## 5 Preventive Measures

### 5.1 Antioxidants

ErbB2-targeting drugs are widely used and an increasing measures for prevention and treatment of cardiotoxicity are being investigated (Dias et al., 2016). Prophylaxis of the antioxidant Probucol (Prob) resulted in a 50% reduction in trastuzumab-treated mice with no significant reduction in left ventricular size or contraction parameters (Walker et al., 2011). In addition, renorizine can also inactivate the cardiotoxicity of trastuzumab by inhibiting the accumulation of ROS through redox-mediated mechanisms. Renolazine also reduced the side effects of pertuzumab and TDM1 (De Lorenzo et al., 2018). Surprisingly, dietary supplementation of flaxseed (FLX), alpha-linolenic acid (ALA), and secoisolariciresinol diglucoside (SDG) also appeared to have cardioprotective effects (Asselin et al., 2020). Similarly, the incorporation of the antioxidant coenzyme Q (10) into nanoemulsion (NES) reduced the expression of leukotriene B4 and p65/nuclear factor-kappa B (NF-κB) and the production of interleukin-1β and interleukin-6 to protect the heart (Quagliariello et al., 2020).

### 5.2 ACEIs/ARBs and BB

The combination of ACEIs/ARBs and BB (ACE inhibitors, angiotensin II receptor blockers and beta-blockers) with trastuzumab adjuvant therapy is beneficial for LVEF recovery (Oliva et al., 2012). ACEI/ARB can change the neurohumoral renin-angiotensin-aldosterone system (RAAS) pathway and prevent heart remodelling. Beta-blockers reduce sympathetic dysfunction (Elghazawy et al., 2020). However, metoprolol had no effect on the overall decline in LVEF (Gulati et al., 2016). It cannot prevent the decrease in LVEF, nor can it prevent severe heart atrophy, heart necrosis, or heart remodelling caused by chemotherapy (Nicol et al., 2021). Beta-1 adrenergic blockade may inhibit sirtuin-3 activation and promote oxidative stress, reducing the protective effect of the sirtuin-3 pathway on mitochondrial function and fibrosis (Guglin et al., 2019). Lisinopril or carvedilol was used to minimize the interruption of trastuzumab. Further prospective studies are required to verify whether this prophylaxis prevents trastuzumab-related cardiac toxicity.

### 5.3 Statins

Statins reduce the risk of heart failure due to anthracycline (Seicean et al., 2012). A recent retrospective study found that statins also reduced the decline in LVEF caused by trastuzumab. A total of 129 patients with HER2-positive breast cancer were treated with desuximab. Forty-three patients were treated with statins during the cancer treatment. The median trastuzumab exposure time was 11.8 months (range, 11–12 months). Compared with the control group, the adjusted final LVEF was lower during a median cardiac follow-up of 11 months (IQR 9–18 months) (61.2 versus 64.6%, *p* = 0.034) (Calvillo-Argüelles et al., 2019). Statins reduce the risk of heart failure after chemotherapy for early breast cancer (including anthracyclines), but the risk associated with the use of statins after trastuzumab treatment remains unclear. Currently, the mechanism underlying the effect of statins on ErbB2-targeted cardiotoxicity remains unclear (Abdel-Qadir et al., 2021). However, one trial found that rosuvastatin inactivates the deterioration of left ventricular function and the production of reactive oxygen species (ROS) and glutathione. Therefore, the pleiotropic effects of HMG-CoA reductase inhibitors may be related (Kabel and Elkhoely, 2017).

### 5.4 Others

In addition, various other methods have been reported to reduce cardiotoxicity. Cardiac monitoring in patients receiving ErbB2-targeted therapy should be a priority (Henry et al., 2018). Monitoring of LVEF for 3 months was considered mandatory (Visser et al., 2016). Strict adherence to guidelines is necessary to avoid serious cardiovascular events. SAFE-HEaRt, a long-term follow-up study, found that continued multidisciplinary care of patients with cancer and heart disease was essential to improve patient outcomes (Khoury et al., 2021). In addition, there was no significant difference in the incidence of adverse events between subcutaneous and intravenous administration, and it was safe and tolerable in HER2-positive early/locally advanced breast cancer (EBC/LABC) (Zambetti et al., 1990; De Sanctis et al., 2021). Surprisingly, moderate-intensity exercise training in patients prevented LVEF and loss of strength (Hojan et al., 13797) (Table 3).

**TABLE 3:** Summary of intervening measure. Current interventions are mainly antioxidants, ACER/AEB/BB and new material combination drugs. Antioxidants are made up of Probucol, Ranolazine, flaxseed (FLX), alpha-linolenic acid (ALA), secoisolariciresinol diglucoside (SDG), the antioxidant coenzyme Q (10) and nanoemulsion (NES). The possible mechanism is mainly antioxidant effect. The drugs mainly affected reactive oxygen species (ROS) accumulation to inhibit of cell death.

Types	Drugs	Test subjects	Function	References
Antioxidant	Probucol	Rats	ROS↓/Myocardial remodeling↓	PMID:21353471
	Ranolazine	Mice	MMP2/Capase3↓	PMID:29467663
	FLX/ALA/SDG	Mice	Antioxidant	PMID: 32510147
	Q(10)+NEs	Humans	LTB4/NF-κB/IL-6↓	PMID: 32764923
ACER/ARB/BB	ACEI/ARB	—	RASS↓/Myocardial remodeling↓	PMID: 32777728
	BB	—	Sympathetic nervous↓	PMID: 32777728
	BB	Mice	Sirtuin-3↓/ROS↓	PMID: 33529501
	Statin	—	GS-SG↓/ROS↓	PMID: 28622591
	Lipidosome	Cells/Humans	Cardiotoxicity↓	PMID: 26759238

## 6 Discussion

ErbB2-targeted drugs cause cardiac dysfunction that is exacerbated when combined with anthracycline chemotherapy for HER2-positive breast cancer. Although cardiotoxicity causes ultrastructural damage to the myocardium, the death of cardiomyocytes was found pathologically. Currently, the mechanism of cardiotoxicity remains unclear. However, the targeted drugs lead to an increase in mitochondrial ROS, activation of endogenous apoptotic procedures, and inhibition of NGR1/HER-2 affecting downstream PI3K/Akt and Ras/MAPK pathways which provide possible explanations for the clinical protective effect of antioxidant drugs. MMP2 is a newly discovered target, which is mainly related to apoptosis. This is similar to the previous discovery of the BCL pathway. Myocardial toxicity and cell apoptosis deserve further study. These molecular structures may be potential therapeutic targets. Current interventions mainly include antioxidants, ACER/ARB/BB, and new material combination drugs. This protective effect may be achieved by reducing the accumulation of mitochondrial ROS. They have been shown to reduce the risk of cardiotoxicity in clinical and animal studies as antioxidants, but there is no further evidence of their roles in the ErbB2-induced pathway. More experiments are needed to verify whether these drugs act on the ErbB2-induced pathway. The main clinical symptom is decreased LVEF, but right ventricular dysfunction has also been reported, and the type of damage caused by cardiotoxicity to cardiomyocytes still further investigation. Risk factors significantly associated with the development of cardiac events were baseline LVEF <60%, hypertension, body mass index >25, age ≥60, and non-Caucasian ethnicity. They are the easiest and most significant indicators for assessing cardiotoxicity before the use of targeted drugs. Whether used alone or in combination, trastuzumab has more severe cardiotoxicity in ErbB2-targeted drugs than other drugs. While pertuzumab, margetuximab, antibody-drug conjugates, and tyrosine kinase inhibitors show less cardiotoxicity, other side effects like diarrhoea, rashes, and blood problems can also be a barrier to taking the medicine. Of course, high cost is also one of the factors that keeps patients away. The cardiotoxicity of trastuzumab combined with pertuzumab is low, but the cardiotoxicity of trastuzumab combined with lapatinib is higher. It is possible that lapatinib directly inhibits ErbB2 phosphorylation, while trastuzumab and pertuzumab have similar ErbB2 epitope-binding sites. Routine detection of LVEF and early presentation of GLS with ErbB2-targeted therapy can predict the development of cardiotoxicity. NT-proBNP has always been a hot marker in the predictive diagnosis of myocardial toxicity. Although there are still contradictory results in all tumor myocardial toxicity studies, it has good specificity and sensitivity in the treatment of breast cancer resulting in myocardial toxicity. NT-proBNP may be used to monitor TIC during treatment and has a predictive effect on TIC prognosis. It is non-invasive, inexpensive, reproducible, and worthy of the attention of clinicians. NT-proBNP may serve as a biological marker for clinical prediction of the occurrence of cardiotoxicity.
